# Engineering of High‐Yield Recombinant Adeno‐Associated Virus Producer Plasmids

**DOI:** 10.1002/biot.70179

**Published:** 2026-01-14

**Authors:** Marco T. Radukic, Dinh To Le, Robert Freudenberg, Anne Hammann, Omar Hamdan, Claire Rothschild‐Gronau, Raimund Hoffrogge, Susanne K. Golm, Rebecca C. Feiner, Kathrin E. Teschner, Kristian M. Müller

**Affiliations:** ^1^ Cellular and Molecular Biotechnology, Faculty of Technology Bielefeld University Bielefeld Germany; ^2^ Center for Biotechnology (CeBiTec) Bielefeld University Bielefeld Germany

**Keywords:** Ad5 helper plasmid, adeno‐associated virus (AAV), membrane‐associated accessory protein (MAAP), plasmid engineering, replicase (Rep)

## Abstract

Recombinant Aadeno‐associated virus (rAAV) production lags demand with respect to quality and quantity. We report insights from producer plasmid engineering aimed at increasing yield and homogeneity of rAAV vectors obtained by HEK‐293 triple transfection. Miniaturized production and same‐day quantification streamlined the investigation. We demonstrate that modifications of the AAV2 Rep gene cluster reduces titers of currently circulating packaging plasmids. Revertants to wild type yielded 116‐fold higher titers of about 10^6^ particles per cell and reduced mispackaging. Modifications of predicted Rep post‐translational modification sites decreased the empty capsid titer burden. A 7 kbp minimal helper plasmid lacking L4 22k maintained production capability upon optimized Rep expression for AAV2 but not for AAV6 and AAV9. Knockout of the egress protein MAAP increased rAAV yield from the cell pellet for convenient lysate processing. Together, these findings highlight the importance and potential tradeoffs of designing producer plasmids to obtain high titer systems.

## Graphical Abstract and Lay Summary

Recombinant adeno‐associated viruses (AAV) are important in gene therapy but their production may be impaired by non‐optimized gene design. In this study, the authors demonstrate that production was best when the AAV replicase gene was in a wild‐type configuration while the complexity of other genetic elements could be reduced.

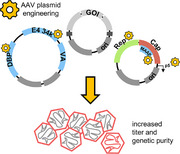

**Abbreviations**
Ad5: adenovirus serotype 5
DBP: Ad5 DNA‐binding protein
ITR: inverted terminal repeat
kbp: kilobasepairs
MAAP: membrane‐associated accessory protein
PTM: post‐translational modification
rAAV: recombinant adeno‐associated virus

## 1 Introduction

Gene therapy based on recombinant adeno‐associated virus (rAAV) vectors has had significant clinical impact with the approval of several therapeutics that are transforming patients' lives. Despite these advancements, rAAV production still faces challenges related to yield and product homogeneity, which are critical for the scalability and affordability of gene therapies. Historically, the development of AAV vectors has been marked by overcoming initial hurdles in vector design and production methods. Early efforts focused on understanding the AAV life cycle and optimizing transient transfection systems, which remain the predominant method for rAAV production today. These systems typically involve producer cell lines transfected with plasmids containing essential genetic elements: the wild‐type AAV genome stripped of its inverted terminal repeats (ITRs), a vector genome cassette flanked by ITRs for packaging, and helper genes from human adenovirus serotype 5 (Ad5) [1, 2]. However, the genetic configurations of these elements, often established decades ago, may impose limitations on rAAV production today.

We have recently published one such example of overcoming historic burden regarding the integrity of the ITRs, which have a high likelihood of being truncated on producer plasmids. We showed that intact and full‐length wildtype ITRs improved rAAV yield and purity in our test system [3]. Here, we report a collection of rAAV production analyses and learnings, obtained over a longer time period in our lab, and representing a considerable evolution of our production system, concerning modifications of the Rep gene cluster, the helper genes of Ad5 and the membrane‐associated accessory protein (MAAP) of AAV.

The Rep gene cluster plays a crucial role in the life cycle of AAV, including the production of recombinant AAV. It comprises four Rep proteins Rep40, Rep52, Rep68, and Rep78, transcribed from a single in‐frame cluster. Rep40/52 are transcribed from the p19 promoter, and the proteins contain a central helicase domain implicated in viral genome packaging, while Rep68/78 mRNA is transcribed from the p5 promoter and the proteins additionally contain N‐terminally a specific nuclease domain necessary for viral genome replication. Alternative splicing of a major or minor intron result in two possible C‐termini, which are implicated in host‐cell interactions (for a review and current perspective see [4]). As engineering targets, Rep proteins are relatively underexplored, probably owing to their complex biology, showcased by their myriads of described host–cell interactions and elusive oligomerization states. Previous studies have, for example, investigated single amino acid substitutions [5] or conducted Rep directed evolution [6] with moderate success in improving rAAV productivity. In this study, we focused attention on possible sites of Rep post‐translational modifications and historic alterations to the Rep gene cluster to investigate production impacts. Very limited data are available, but differential ubiquitination of Rep depending on helper virus presence has been described [7], although no specific lysine residue for ubiquitination was identified. Additionally, SUMOylation of Rep could occur at K84 [8]. Here, we aimed to investigate the impact of these sites on rAAV production in terms of viral genomes (vg) per transfected cell, but also in terms of capsid titer, since the cap p40 promoter is located within the Rep coding sequence and such data are currently not available.

Next to the Rep genes, the plasmid‐provided helper genes derived from human adenovirus serotype 5 (Ad5) are typically used in the production of rAAV. Recent studies have reported so‐called minimal sets of Ad5 helper genes [9, 10]. Older studies showed that cellular stress factors like genotoxic agents, UV light, or cell extracts principally suffice to sequester the cell into AAV production [11, 12, 13], posing the question if a strict set of helper genes exists. Previous reports did not explore minimal helper sets in terms of an AAV productivity per cell. However, the utility of a mini helper system may be dependent on the productivity of the overall system, potentially showing an advantage only in a particular production context. Therefore, we aimed at investigating a minimal helper set comprised of the Ad5 DNA‐binding protein (DBP), E4orf6 protein, and VA RNA I + II in context of our known high‐producer system in terms of a cell‐specific yield.

A further potential target to engineer high titer rAAV plasmids is the Membrane‐Associated Accessory Protein (MAAP) which emerged as another intriguing option for producer plasmid engineering. The protein has been described as an egress factor for AAV from the producer cell [14]. We, therefore, investigated the knock‐out of MAAP as a potential strategy to improve AAV production, since many published production processes utilize cleared cell lysate for downstream processing.

We supported our analyses by development of a miniaturized screening assay to compare iterations of producer plasmids efficiently. The assay is based on miniaturization of rAAV production in multiwell plates and in‐lysate titration enabled by the use of a salt‐active nuclease.

Together, a multifaceted approach to engineering high titer rAAV producer plasmids is presented, supported by a down‐scaled, parallelized approach to compare productions.

## 2 Materials and Methods

All standard reagents were purchased in analysis grade from Roth or Merck/EMD. Cell culture media and additives were purchased as cell‐culture grade from Merck. Laboratory plastic consumables were purchased from Sarstedt. Desalted oligonucleotides for cloning procedures were purchased from Merck. Oligonucleotides for qPCR were purchased in HPLC‐grade from Merck. Enzymes for cloning and analysis were purchased from NEB. Plasmid DNA was prepared using mini‐ and midi‐prep Kits obtained from Macherey–Nagel (obtained OD_260_/OD_280_ within a range of 1.83–1.88).

### 2.1 Plasmids

Requests for plasmids used in this study should be directed to the corresponding author. The starting point for our study was a three‐plasmid system based on pHelper (pZMB0088, Agilent catalog #240071, “AAV Helper‐Free System”), a pRep2Cap2 provided by iGEM team Freiburg 2010 (“pSB1C3_RepVP123_p5TATAless”) based on pAAV‐RC (Agilent) and an ITR plasmid encoding a 119 bp and a 130 bp ITR and mVenus transgene (pZMB0522). Plasmids used in this work are listed in Table 1.

**TABLE 1:** Plasmids used in this study.

Lab. ref.	Description	Source
pZMB0088	Adenoviral helper functions	Agilent
pZMB0216	Rep2 Cap2 encoding plasmid	[15, 16], iGEM Freiburg 2010
pZMB0430	See supplementary sequence S1. Like pZMB216 with added XmaI‐site (overlap‐extension PCR) downstream of the Rep CDS though a silent Cap mutation to facilitate cloning of Rep variants; introduces a L22P mutation in MAAP, progenitor to plasmids pZMB0431 – pZMB0440	This work
pZMB0431	Rep substitution variant P2A, K33A (variants 431 – 440 are cloned by overlap‐extension PCR on pZMB0430)	This work
pZMB0432	Rep substitution variant P2A, K84R	This work
pZMB0433	Rep substitution variant P2A, K204A	This work
pZMB0434	Rep substitution variant P2A, K506R	This work
pZMB0435	Rep variant P2A, N525* (stop codon TAA)	This work
pZMB0436	Rep variant P2A, K533* (stop codon TAA)	This work
pZMB0437	Rep substitution variant P2A, S535A	This work
pZMB0438	Rep variant P2A, 529(17)* (stop codon TGA)	This work
pZMB0439	Rep variant P2A, R532G‐only	This work
pZMB0440	Rep substitution variant P2A, Y224A	This work
pZMB0504	A packaging plasmid encoding Rep2 (Rep configuration as pZMB0818) and Cap9	Unpublished
pZMB0522	ITR plasmid encoding an mVenus fluorescence reporter	[16]
pZMB0574	A packaging plasmid encoding Rep2 (Rep configuration as pZMB0216 but wildtype p5) and Cap6	Unpublished
pZMB0618	Figure 1, variant B	This work
pZMB0630	Figure 1, variant A	This work
pZMB0797	See supplementary sequence S2. pHelper‐mini (compared to an Ad5 reference, GenBank AC_000008, retained parts were for VA‐RNA, roughly nucleotides 10,436 – 11,167, for E4 roughly nucleotides 34,087 – 33,195, and for E2A roughly nucleotides 24,055 – 22,188 with polymorphisms retained from the Agilent pHelper, pZMB0088. The plasmid was ordered as a gene synthesis.)	This work
pZMB0815	E4‐34k‐only helper	This work
pZMB0818	Figure 1, variant C	This work
pZMB0887	Like variant B (pZMB0618), but ΔMAAP	This work
pZMB0916	See supplementary sequence S3. Like variant C (pZMB0818), but ΔMAAP	This work

### 2.2 Packaging Plasmid Meta‐Analysis

A list of 118 plasmid IDs annotated as “AAV packaging plasmids” was obtained from Addgene (https://www.addgene.org/viral‐vectors/aav/, accessed 14.11.2025) and all plasmid sequences were programmatically downloaded using a custom Python script. Sequences were then mapped to the reference AAV2 genome (GenBank AF043303.1) with Geneious (Biomatters/Siemens) and features of interest were manually evaluated (see text). The Rep40/68 N‐terminal sequence overlapping Cap was ignored in this analysis because it typically depends on the Cap serotype. Analyzed Addgene IDs were: 112865, 103005, 112864, 112862, 64839, 110770, 103006, 112866, 112863, 175004, 81070, 110660, 92307, 103002, 183749, 184541, 130878, 64867, 184540, 68837, 110809, 212708, 184539, 200257, 196836, 127851, 197565, 175005, 185137, 170716, 185136, 127847, 65214, 78504, 240485, 205991, 206504, 224440, 218796, 224448, 224687, 220799, 184592, 166921, 192262, 166888, 195218, 65215, 184542, 65216, 184543, 65213, 78503, 240486, 237878, 237877, 236254, 233684, 232922, 232199, 232174, 232173, 224452, 224451, 224450, 224449, 224447, 224446, 224445, 224444, 224443, 224442, 224441, 224439, 224438, 224437, 222333, 218799, 218798, 218797, 213974, 213968, 209780, 206513, 206512, 203536, 203535, 203534, 203533, 203532, 201903, 199746, 199745, 199744, 199599, 198016, 198015, 198014, 196691, 196690, 196689, 196688, 196687, 196686, 196685, 196684, 196683, 196682, 196681, 196680, 196679, 174539, 130877, 129024, 127849, 127848, 103004, 103003.

### 2.3 Down‐Scaled AAV Production

Culture and transfection parameters were down‐scaled according to growth area from previously published protocols [17]. Briefly, HEK‐293 cells (DSMZ) were kept in DMEM, 10% fetal calf serum (Thermo), 1% penicillin/streptomycin, 37°C, 5% CO_2_. For AAV production, 0.51 × 10^6^ cells were seeded per well of 6‐well plates. The next day, cells were transfected by the calcium phosphate method. For standard AAV production, 2.55 µg plasmid DNA in a molar 1:1:1 plasmid ratio (pITR:pRepCap:pHelper) was used. Unless stated otherwise, plasmids for AAV production were lab ref. pZMB0088 (pHelper, Agilent) and pZMB0522 (pITR). DNA was diluted in 85 µL 0.3 M CaCl_2_ solution and then injected into an equal volume of 50 mM HEPES, 1.5 mM Na_2_HPO_4_, and 280 mM NaCl. The obtained mixture was rapidly pipetted up and down ten times and added to the cells. Transfection success was investigated by fluorescence microscopy 3 days post‐transfection and was typically higher than 90%, or the sample was rejected.

### 2.4 Sample Preparation for AAV Titration

Samples from down‐scaled AAV productions were prepared for qPCR titration in one of two ways. Initially, parallel, small‐scale batch purification of AAV was employed. Cells were scraped in their culture media and pelleted (3000 × *g*, 5 min). The supernatant was discarded, and cells were resuspended in 1 mL Tris‐buffered saline (50 mM Tris base, 2 mM MgCl_2_, 150 mM NaCl, pH 8.0). The suspension was subjected to three freeze‐thaw cycles (−80°C, 10 min, to 37°C until thawed). Benzonase nuclease was added to a final concentration of 60 U/ml and incubated (37°C, 1 h). CHAPS was added to 0.5% w/v and incubated (37°C, 30 min). The lysate was then cleared by centrifugation (3000 × *g*, 5 min, 4°C) transferred to a new microcentrifuge tube, and cleared further (20,000 × *g*, 10 min, 4°C). Twenty‐five microliters slurry of affinity resin AAVX (Thermo) was added to the lysate supernatant and incubated on a temperature‐controlled shaker, 10 min, 21°C, 600 RPM, 1 cm amplitude. The slurry was pelleted, 1000 × g, 5 min, and washed in three steps with 600 µL phosphate‐buffered saline (137 mM NaCl, 2.7 mM KCL, 10 mM Na_2_HPO_4_, 1.8 mM KH_2_PO_4_, pH 7.4) for 5 min. For elution, the resin pellet was resuspended in 200 µL glycine‐HCl, pH 2.2, incubated for 3 min, centrifuged, 1000 × *g*, 5 min, and the supernatant was transferred to a fresh vessel and neutralized with a pre‐determined appropriate amount of 2 M Tris‐base to a pH of 7.5–8.0. The neutralized eluate was then stored at −80°C until analysis. The chromatography slurry was subsequently regenerated for future use: Five slurry‐volumes 0.1 M phosphoric acid, 5 min, centrifugation, five volumes 6 M guanidine‐HCl, 5 min, centrifugation, five volumes phosphate‐buffered saline, 5 min, centrifugation, finally, storage, 20% ethanol in water, 4°C.

The method was later adapted for direct in‐lysate titration of AAV to account for a higher sample throughput. Complete digestion of free DNA and proteinogenic PCR‐inhibitors was achieved by use of a salt‐active nuclease and a supporting salt‐active protease. Per transfected well, cells were scraped with a silicone cell scraper and resuspended in Tris‐buffered saline as before. 10 µL of the homogenous cell suspension was then transferred to a PCR tube, 2.64 µL of 2 M NaCl (500 mM final concentration) and 0.96 µL of 50 mM MgCl_2_ (5 mM final concentration) were added, and samples were subjected to three freeze‐thaw cycles (−80°C, 10 min, to 37°C until thawed). One microliter SAN HQ salt‐active nuclease (ArcticZymes) was added and incubated, 37°C, 3 h. 1.62 µL of a 0.5 M EDTA base solution (pH 8.0, final concentration 50 mM) and 1 µL AZ protease (ArcticZymes) was added and further incubated, 37°C, 1 h. The protease was finally inactivated, 95°C, 20 min. Samples were stored frozen and undiluted at −80°C until analysis.

We used equal volumes of enzyme in these tests, due to differences in unit definitions (e.g., DNaseI: 1‐unit digests 1 µg of pBR322 DNA in 10 min at 37°C; Denarase: 1 unit is digestion of salmon sperm DNA to acid‐soluble oligonucleotides equivalent to a ΔA260nm of 1.0 in 30 min at 37°C). Denarase is an amino acid equivalent to Benzonase.

The capsid titer was determined by a commercial capsid ELISA (#PRATV, Progen) as per the manufacturer's protocol.

### 2.5 qPCR and RT‐qPCR

Samples for AAV titration were diluted 100‐fold in water containing 0.005% Pluronic F‐68. SYBR‐green‐based qPCR (GoTaq qPCR, Promega) was then performed with primers targeting the CMV promoter (5′‐GGGACTTTCCTACTTGGCA and 5′‐GGCGGAGTTGTTACGACA) present in the transgene or the beta‐lactamase gene (5′‐CAACTTTATCCGCCTCCATC and 5′‐AAGCCATACCAAACGACGAG) present on all plasmid backbones, as we have previously described [17]. All primers were purchased in HPLC grade.

For RT‐qPCR, RNA was extracted using a kit (Quick‐RNA miniprep, Zymo), including an DNaseI step. Eluted RNA was treated with DNaseI for a second time, since we found AAV ssDNA to be quite DNaseI resistant. In detail, 20 µL RNA eluate was mixed with 2.5 µL DNaseI (RNase‐free, NEB), 2.5 µL 10× DNaseI buffer, and 1 U/µL murine RNase‐inhibitor (NEB), and incubated, 37°C, 30 min, 75°C, 10 min. RNA was investigated on a Tris‐borate‐EDTA agarose gel for integrity. cDNA was written using the LunaScript RT SuperMix kit (NEB) and samples were measured using the same qPCR kit with no‐template and no‐RT (DNA carry‐over) controls always present. RT‐qPCR results for E4 (5′‐ACTACGTCCGGCGTTCCAT and 5′‐GGAGTGCGCCGAGACAAC) and Rep (5′‐CGGAGAAGCAGTGGATCCA and 5′‐ATTTGGGACCGCGAGTTG) were analyzed with the ΔΔCq method with a GAPDH primer pair (5′‐ATTGCCCTCAACGACCACTT and 5′‐AGGTCCACCACCCTGTTGCT) for normalization.

### 2.6 AAV Transduction Assay

Cell lysates were used for AAV transduction assays. Lysate at physiological salt concentrations after freeze‐thaw cycles and clearance by centrifugation (3000 ×*g*, 5 min, 4°C, 20,000 ×*g*, 10 min, 4°C, filtration 0.2 µm) was diluted in Tris‐buffered saline and added to HEK‐293 cells in 24‐well polycarbonate tissue culture plates, 1.02 × 10^5^ cells per well seeded 24 h prior to transduction. The transduction efficiency was determined after 3 days using a flow cytometer (BD Accuri C6 Plus), gating to 1% false‐positive fluorescence relative to an untransfected control. Data were analyzed by fitting a four‐parameter logistic curve by the least sum of squares approximation method.

### 2.7 Western Blot Analysis

SDS‐gels were transferred to semi‐dry blotting buffer (25 mM Tris, 192 mM glycine, 20% (v/v) ethanol, pH 8.2) and incubated for 10 min. Proteins were blotted onto a nitrocellulose membrane (0.45 µm, Thermo) at 4 mA per cm^2^ gel for 30 min. Transfer success was determined with Ponceau S solution (0.1% (w/v) Ponceau S, 5% (v/v) acetic acid) for 10 min, and de‐staining for 10 min in Tris‐buffered saline—TBS (50 mM Tris, 150 mM NaCl, pH 7.6). The membrane was blocked with 3% BSA in TBS for 1 h. Primary anti‐replicase antibody 226.7 (100 µL per staining, anti‐AAV2 Replicase mouse monoclonal, 226.7, Progen #65172) in 5 mL blocking buffer was incubated for 1 h, RT. After three washing steps with TBS‐T (TBS, 0.05% v/v Tween‐20), the secondary anti‐mouse peroxidase‐conjugated antibody was applied (1:2500, Thermo #31430), 1 h, RT. Finally, the membrane was washed four times, 10 min, in TBS‐T and developed with peroxidase substrate (SuperSignal West Pico PLUS, Thermo) and imaged (Phusion FX imager, Vilber).

### 2.8 Next Generation Sequencing

Long read nanopore sequencing and data analysis was conducted essentially as described [17]. Briefly, AAVs were purified by one‐step affinity chromatography and packaged DNA was extracted. Purified AAV DNA was tagmented (SQK‐RAD004) and sequenced on R9.4.1 flow cells. Reads were then mapped and assigned to the transgene, backbone, RepCap or helper sequences obtained from the respective producer plasmids.

### 2.9 Nano‐Scale Liquid Chromatography‐Tandem Mass Spectrometry

Nuclear and cytosolic fractions were generated and prepared with the Filter‐Aided Sample Preparation (FASP) method for nano‐scale liquid chromatography‐tandem mass spectrometry (nLC‐Orbitrap MS/MS) as recently described by us [18]. The human protein database (Homosapiens SwissProt DB, TaxID 960, version October 25, 2017) and the sequences of the AAV proteins translated in silico from the respective plasmids used for AAV production were provided as reference in Proteome Discoverer software 3.0 (Thermo).

## 3 Results

### 3.1 AAV2 Rep Gene Alterations in Packaging Plasmids Are Widespread in the Academic Research Community

We took the Rep gene as a starting point to investigate production bottlenecks and compared our initial packaging plasmid (pZMB0216) to the wild‐type sequence (GenBank AF043303.1). This plasmid carried changes in the Rep78/52 Kozak sequence, an associated proline to alanine mutation (P2A), and was missing a TATA box in the p5 promoter. The TATA‐less p5 promoter was previously patented (pHLP19 with p5 Δ TATA) but little data on the production efficacy were given [19]. In addition, the promoter was positioned at the end of the Rep Cap region, thus transcription and translation start are interspersed by the plasmid backbone, which is also a patented strategy to reduce rep expression [20]. These promoter modifications were incorporated in one of the first commercially successful AAV plasmid collections (Agilent catalog #240071, “AAV Helper‐Free System”).

A meta‐analysis of currently distributed packaging plasmids revealed that Rep gene alterations are widespread. Of 118 “AAV packaging plasmids” available to researchers through Addgene, only 25 plasmids (21%) encoded the wildtype Rep78 amino acid sequence, with a P2A substitution (*n* = 10) and substitutions in the Rep68/40 intronic region (*n* = 39) being particularly common. At least four of these plasmids were meant for a baculovirus production. 19 plasmids (16%) did not encode the canonical start codon AUG, but a weak start codon ACG or CUG. One plasmid did not have the p5 TATA box, while five plasmids encoded a truncated p5 (referring to a truncation of nt 190–310 of the reference sequence). The p5 promoter was either located downstream of Cap, essentially upstream of the backbone (*n* = 61, 52%), or located directly upstream of Rep (*n* = 48, 41%), while there appeared to be no correlation between the location of p5 and usage of a weak start codon. These changes to Rep were typically not discussed in an investigated subset of the respective original manuscripts referenced by Addgene (eight of ten papers did not discuss Rep changes or referenced a source for the alterations). These findings show that Rep gene variants are common in the community and warrant to further investigate the influence of alterations on rAAV productivity in a current production context.

### 3.2 A Wild‐Type‐Like Rep Gene Drives High Titer AAV Production

**FIGURE 1:**
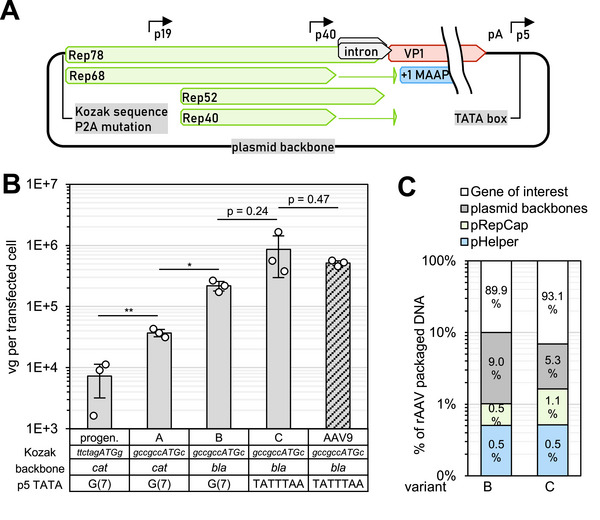
Influence of Rep gene modifications on rAAV production. (**A)** Scheme of investigated alterations in the p5 promoter and Rep Kozak sequence, including a P2A mutation. A different plasmid backbone was also investigated. Progenitor: pZMB0216. (**B)** Reverting previous alterations back to AAV2 wild‐type dramatically improved rAAV production. (**C)** Rapid Nanopore sequencing revealed a decreased impurity profile for variant C, which is closest to a wild‐type genome. *p < 0.05, **p < 0.01. Error bars show the standard deviation (SD) of biological replicates (circles).

We addressed alterations in our packaging plasmid (Figure 1A) by stepwise reverting them back to the wild type sequence. Switching to a different pRepCap plasmid backbone was also investigated in this context. In all tested cases, we observed remarkable increases in rAAV genome titer (Figure 1B). The reversion of Rep68/78 Kozak sequence including the alanine (codon GCG) at position 2 to the wild‐type proline (codon CCG) increased the AAV titer 5‐fold compared to the progenitor. Switching the plasmid backbone from pSB1C3 to pUC increased AAV yield 6.7‐fold. Since pSB1C3 is also a pUC derivative, the main change relates to the replacement of a *cat* gene coding to chloramphenicol acetyltrasferase resistance to a *bla* gene coding for beta‐lactamase. These bacterial genes are not per se controlled by an eukaryotic promoter but are sandwiched between the p5 promoter and the Rep/Cap cassette in our system. Since the transcription start site is within the p5 promoter [21] upstream of the bacterial sequences, these sequences may influence mRNA stability and, thus, Rep expression. In addition, the number of potentially skipped start codons is similar between the pSB1C3 backbone (22 ATG skipped) and the pUC backbone (24 ATG skipped) and the 5′ UTR would be roughly 2 and 2.8 kbp long for pSB1C3 and pUC‐based variants, respectively. Finally, re‐installing the wild‐type TATA‐box in TATA‐less p5 promoter increased the AAV titer further 3.5‐fold. In total, the AAV titer increased 116‐fold compared to the progenitor plasmid.

We also analyzed the genetic homogeneity of the resulting rAAV produced from the high titer variants (labeled “B” and “C” in Figure 3B) using our established direct sequencing workflow [17] and found that the overall genetic homogeneity of the AAV packaged DNA increased with titer (Figure 1C) while mispackaging of RepCap sequences increased with the TATA‐wildtype variant. In our previous study, RepCap sequences were packaged from a well‐known alternative packaging signal in the p5 promoter [17]. It may, thus, be necessary to carefully weigh high titer production against p5‐mediated mispackaging.

Interestingly, the final titer was highly similar to that of an rAAV9 production from a similar AAV2‐Rep configuration (Figure 1B), suggesting that AAV2 is, in fact, not a “low producer” variant, as is still sometimes quoted, when Rep is properly configured for the respective production system. Since many AAV RepCap plasmids with Cap of different serotypes utilize Rep of AAV2, it is likely that our results are transferrable to other serotypes as well.

### 3.3 Rep Gene Variants May Tailor Capsid Expression to Genome Replication

**FIGURE 2:**
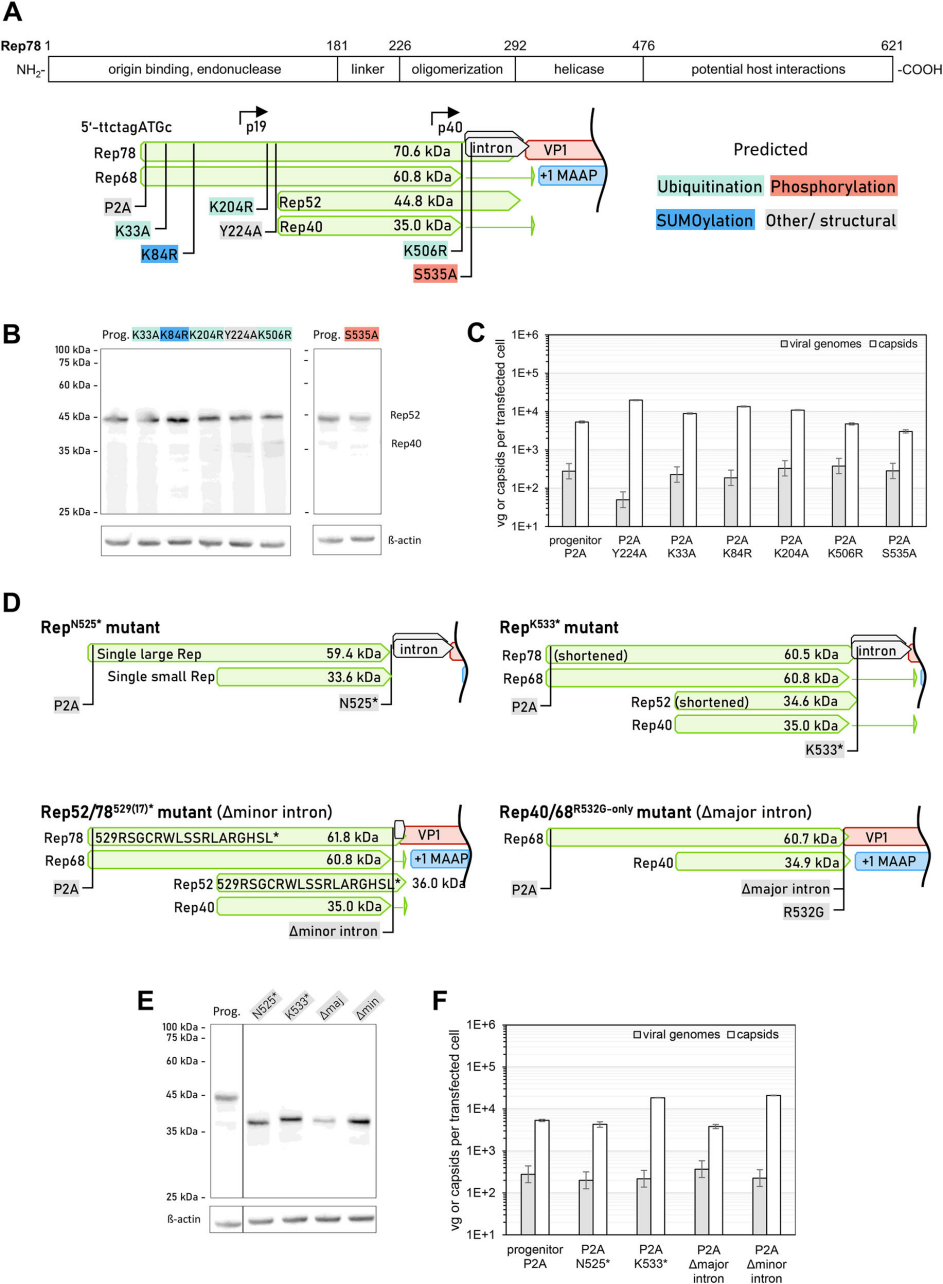
Investigation of Rep gene variant impact on rAAV production. (**A)** Linear sequence scheme of the Rep78 protein (top) and map of investigated single amino acid substitution variants on a P2A background (bottom). Predicted sites of post translational modifications are color coded. (**B)** Anti‐Rep Western blot analysis of the variants of A. Progenitor was pZMB0430. (**C)** Viral genome and capsid titer of tested variants. (**D)** Map of investigated structural variants. Deletion of the minor intron leads to addition of 17 amino acids to the Rep52/78 C‐termini. (**E)** Anti‐Rep Western Blot of the variants of D. (**F)** Viral genome and capsid titer of tested variants. Error bars are standard deviations from two technical replicates.

We further investigated the influence on production of rationally designed variants of the Rep proteins and of the genetic elements of the Rep gene in context of the progenitor plasmid pZMB0430 (pZMB0216 from Figure 1, “progenitor”, with added XmaI site in Cap through a silent mutation). Unknown to us at the time of experimentation, the addition of the XmaI site added a L22P substitution in MAAP, which all further plasmids in this chapter retain. Firstly, we generated, on DNA level, five conservative amino acid substitution mutants of Rep in the pRepCap context that erase potential sites of post‐translational modifications (PTMs) like ubiquitination, phosphorylation, and SUMOylation, since PTMs often influence protein turnover, localization, function, and interactions, which in‐turn may influence rAAV production (Figure 2A for scheme of mutations and their role). Sites of possible ubiquitination were predicted using UbPred [22]. The SUMOylation site was taken from literature [8]. A S535A mutation was reported to increase viral genome replication, but its effect on overall production was not investigated [23]. In addition, we used a known loss‐of‐function variant, Y224A, to establish a dynamic range of the productivity [24]. Investigated sites were located within the origin binding and endonuclease domain (K33A, K84R), the linker (K204R, Y224A), and the largely unstructured C‐terminal part (K506R, S535A). In case of lysine positions with potential structural impact (K204R, K506R), a conservative conversion to arginine was chosen. In addition, all mutants harbored the described proline to alanine substitution at position 2.

Western Blot analysis showed that all tested Rep amino acid substitution variants expressed similar amounts of Rep proteins, with Rep52 being the dominant form (Figure 2B), while the large Rep proteins were mostly undetectable. Rep40 was probably present with up to three isoforms, potentially from improper termination. None of the tested mutants significantly influenced the genomic titer, except for the known loss‐of‐function mutation Y224A (Figure 2C, grey bars). However, capsid titers were significantly up‐ or downregulated (Figure 2C, white bars), indicating that it is possible to control the empty capsid load of a production run by introducing such changes to the plasmid system. Variant Rep78/68 S535A substitution showed the most pronounced reduction in capsid titer, while maintaining the viral genome titer.

We also generated deletion variants of the Rep introns (Figure 2D), which are located downstream of the helicase domain and provide variation in the Rep C‐terminus. The Rep C‐terminal domain is described as unstructured without participation in genome replication or packaging [25]. Variant N525* terminated all four Rep proteins before the introns, effectively resulting in only two Rep proteins. Variant K533* terminated only the large Rep proteins, leaving Rep40 and Rep68 unaffected. Deletions of the minor and major intron maintained the respective C‐terminal amino acids that overlap with Cap.

Again, Rep52 or the respective small Rep variant was dominantly expressed (Figure 2E) and none of the variants influenced the viral genome titer, while the capsid titer varied (Figure 2F). Interestingly, the capsid titer was lower compared to the progenitor plasmid when only a single small and a single large Rep was expressed, while the capsid titer increased dramatically when the C‐termini of Rep78 and Rep52 were truncated, although it remains to be elucidated if these changes are caused by Rep protein or by changes to the gene structure, which includes the Cap p19 promoter. These findings show how Rep engineering may be used to minimize the empty capsid load of a production. In addition, a potentially simplified production system with only a single large and a single small Rep is feasible.

Overall, since a productivity approaching 10^6^ vg/cell already surpasses many wildtype viruses and engineering efforts by us and others largely did not further improve productivity, the combined findings suggest that the virus titer in the current production system is not limited by wildtype Rep. Still, Rep is an interesting engineering target to tune the genome to capsid ratio. Conversely, our findings highlight the need to closely inspect the Rep gene on each producer plasmid, even plasmids for Cap serotypes other than serotype 2, for possible alterations that could influence productivity in the context of the respective production system, when productivity is unexpectedly low.

### 3.4 Adenoviral Helper Genes DBP and E4 34k Suffice for High‐Titer AAV2 Production

The pHelper plasmid, which encodes adenoviral elements E4, E2a, and VA‐RNA, is the largest plasmid in the three‐plasmid system and makes up roughly half of the DNA mass needed for transfection‐based AAV production. Reducing the pHelper size could, thus, offer a further advantage in rAAV production and/or a cost advantage, since less DNA would be needed for transfection.

**FIGURE 3:**
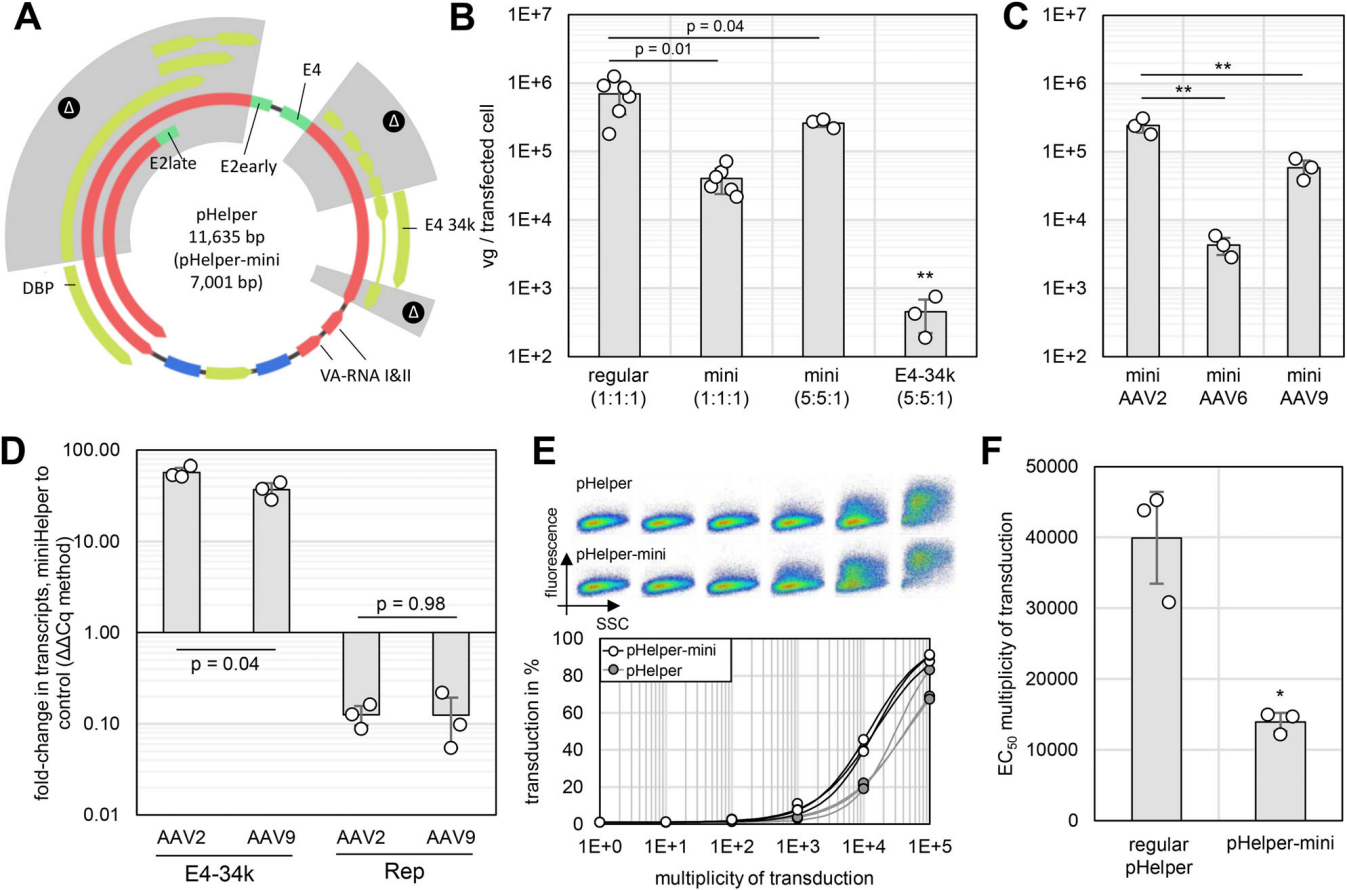
Engineering of a pHelper‐mini. (**A)** Plasmid map of the progenitor pHelper with promoters (dark green), primary transcript regions (red), deleted regions (gray shading, marked with Δ), and transcripts (light green)). (**B)** Comparison of pHelper‐mini and the progenitor for AAV production with optimized AAV2 packaging plasmid pZMB818 (variant “C”, Figure 1). Molar ratios are pRepCap:pITR:pHelper‐mini. A variant of pHelper‐mini only encoding E4 34k was also tested. (**C)** Test pf pHelper‐mini with different serotypes. The AAV9 packaging (pZMB0504) plasmid has a comparable Rep configuration to the AAV2 plasmid (pZMB0818), but the AAV6 packaging plasmid (pZMB0574) misses the Rep Kozak sequence. (**D)** RT‐qPCR analysis of E4 34k and Rep transcription. GAPDH was used as the housekeeping gene. All changes to control are significant. (**E)** Transduction tests of HT1080 cells with AAV2 produced with pHelper‐mini or the regular pHelper and flow cytometry readout. Top: Exemplary dot plots from flow cytometry. Bottom: Resulting response curve with four‐parameter logistic fit. (**F)** EC_50_ comparison of the fit of E. EC_50_ is the concentration required to transduce half of the cell population. *p < 0.05, **p < 0.01. Error bars show SD of biological replicates (circles).

To create such a miniaturized helper plasmid, we designed a plasmid based on the adenovirus type 5 reference genome (GenBank AC_000008), inspired by a typical current large pHelper (Agilent), in which we removed all sequences of the E2a gene that were not promoter or downstream oriented coding sequences, leaving only DBP. For the E4 gene, we removed all coding sequences except for E4orf6 protein E4 34k (Figure 3A). We termed the new helper plasmid pHelper‐mini, which was ordered as gene syntheses and is only 7.0 kbp instead of 11.6 kbp in size (Figure 3A).

We found that pHelper‐mini initially produced less rAAV2 compared to the unmodified pHelper in context of the optimized pRepCap plasmid (variant “C” of Figure 1) when the total transfected DNA mass per cell and molar plasmid ratios were maintained (Figure 3B). Productivity was largely rescued with transfection optimization (0.37‐fold change to progenitor, *p* = 0.04) at a molar 5:5:1 ratio (pRepCap:pITR:pHelper‐mini) at constant transfected mass. An even smaller variant of pHelper‐mini only encoding E4 34k was not able to rescue production. pHelper‐mini was also tested with an AAV9 packaging plasmid offering the same Rep context, and an AAV6 packaging plasmid more comparable in terms of Rep to our unoptimized progenitor. The resulting titer was lower compared to AAV2 in both cases, suggesting that pHelper‐mini is specific to AAV2 (Figure 3C).

RT‐qPCR on the E4orf6 and Rep transcripts in transfected cells (molar 1:1:1 ratio) in AAV2 and comparable AAV9 production context showed that the E4orf6 transcript is significantly upregulated in the pHelper‐mini‐based production compared to a regular pHelper (Figure 3D) at the unoptimized ratio of 1:1:1, while Rep is downregulated. We suspect that removal of the other reading frames in the E4 gene probably leads to E4 34k overexpression. Since E4 34k is known to degrade Rep [26], a reduction in rAAV titer could be expected from E4 34k overexpression and the reduction in gene dose after transfection optimization probably counteracts this effect. Contrarily, the large pHelper progenitor probably does not require this optimization because the added DNA “baggage” already reduces E4orf6 transcription for an optimal balance with Rep.

Interestingly, rAAV2 produced with pHelper‐mini outperformed rAAV2 produced with the progenitor in a transduction test on HT1080 cells, with a significant 0.17‐fold change in EC_50_ (Figure 3E,F). These findings underline the necessity to carefully tune the expression of components, especially of Rep.

### 3.5 Deleting MAAP Increases AAV2 Yield From the Cell Pellet

**FIGURE 4:**
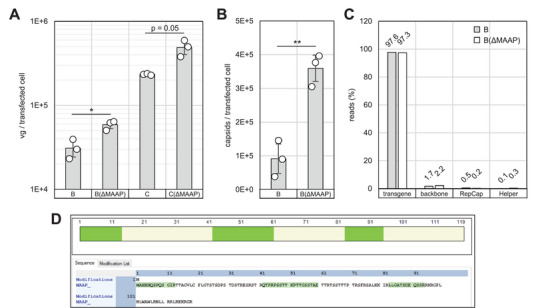
Investigation of MAAP knock‐out on AAV production in context of a regular pHelper pZMB0088 and vector plasmid pZMB0522. (A) Packaged genomes per transfected cell. Variants B and C are as in Figure 2. B(ΔMAAP) is pZMB0887, C(ΔMAAP) is pZMB0916. (B) Capsids per transfected cell of variant B and B(ΔMAAP). (C) Percentage of sequencing reads mapping to either the transgene, plasmid backbone, RepCap or Helper plasmids. (D) Screenshot from Protein Discoverer highlighting unique peptides identified in wt MAAP AAV producing cells but not in ΔMAAP samples. *p < 0.05, **p < 0.01. Error bars show SD of biological replicates (circles).

We additionally investigated the impact of MAAP for AAV2 production in line with our pHelper‐mini and Rep efforts to elucidate and reduce the complexity of the production system. Mutation of the MAAP start codon from CTG (Met/Leu) to CGG (Arg) in context of the high‐titer packaging plasmid variants “B” (resulting in pZMB0887) and “C” (resulting in pZMB0916), which does not affect the coding of the VP1 in the other reading frame, led to an approximately 4‐fold increase in capsid titer and a 2‐fold increase in viral genome titer compared to the best performing Rep gene variant “C” (Figure 4A,B). Long‐read sequencing of the packaged DNA content suggested comparability between the variants and no reduction in AAV quality from the MAAP knock‐out (Figure 4C).

Nano‐scale liquid chromatography‐tandem mass spectrometry analysis after tryptic digest of the cytosolic and nuclear protein fractions of AAV producing HEK‐293 cells (three biologic replicates) showed that in MAAP knock‐out samples (pRepCap lab ref. pZMB0916) no unique peptides of MAAP were identified. In contrast, MAAP (pRepCap lab ref. pZMB0618) was confidently identified in wt MAAP samples with four unique peptides in both fractions, covering 37% of the MAAP sequence (Figure 4D). The result aligns with another study where no MAAP expression was reported when the MAAP start codon was mutated [27]. In contrast, a study by Ogden et al. [28] showed that the mutation of the MAAP start codon can lead to the expression of a truncated MAAP.

MAAP has been described as an excretion factor for AAV and may negatively regulate AAV DNA replication [14, 29]. This could explain the increase of AAV titer observed in the absence or low expression level of truncated MAAP protein. Our finding correlates with the study of complete MAAP knock‐out by mutating of the start codon and introducing of the early stop codon leading to enhanced AAV production [29]. However, others reported that the MAAP deletion has not improved AAV production [27, 30], a discrepancy potentially attributed to differences in AAV production, purification and analysis methods. For example, in this study, the AAV titer was measured in the cell lysate of producer cells only. In addition, the AAV capsid titer of the production with the mutated *maap* was higher than that of wt *maap*. This could be explained by the role of MAAP in inducing VP protein degradation during the production [27].

### 3.6 Processive Nucleases Enable Fast In‐Lysate Quantification of Small‐Scale rAAV Productions

Lastly, we note that throughout our investigation we observed variability in titers from the same plasmids transfected in seemingly comparable experiments. To better evaluate producer plasmid variants for their influence on AAV production while minimizing confounding factors such as differences in cell passaging, we developed an easy to parallelize and rapid rAAV in‐lysate quantification workflow, which enabled robust comparisons. Similar approaches based on recombinant bovine pancreatic DNaseI‐digestion have been described [31, 32], but these require overnight digestion. In contrast, highly processive nucleases are commercially available, which should enable a 1‐day analysis workflow from sample to result.

**FIGURE 5:**
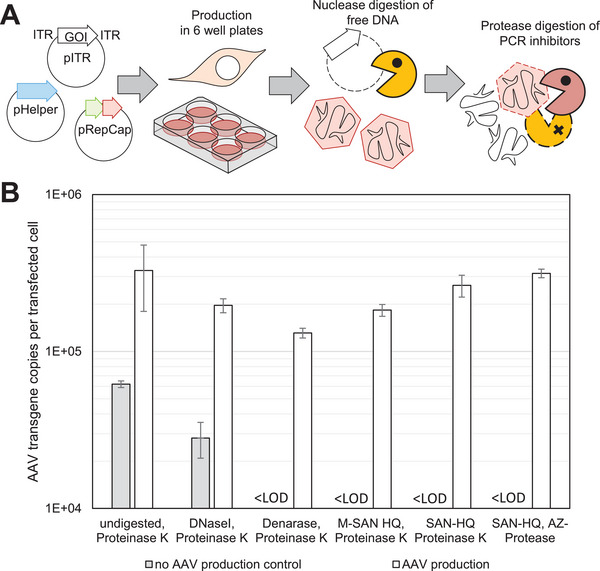
Evaluation of enzymes for in‐lysate AAV quantification. (**A)** Workflow based on transfection of three plasmids for AAV production (left, circles) in 6‐well plates, harvest, adjustment of buffer conditions for the respective nuclease, freeze‐thaw, digestion of free DNA with a nuclease (yellow), and digestion of the nuclease, capsids, and proteinogenic PCR inhibitors with a protease (red). (**B)** qPCR results as transgene copies per transfected cell. Two samples were investigated per nuclease/protease combination and treated equally: A production control without the RepCap plasmid during transfection (grey bars) and an AAV production sample (white bars). Error bars indicate standard deviations from two technical replicates. Differences of the undigested AAV production to nuclease‐digested AAV production samples are not significant, probably due to the large variance in the undigested sample (*p* > 0.05). LOD: Limit of detection, no amplification in qPCR.

We tested three processive nucleases (Denarase (*Serratia marcescens* DNase), M‐SAN HQ (medium salt active nuclease high quality), SAN‐HQ (salt active nuclease high quality) against DNaseI at a constant incubation time of 3 h, with each reaction buffer condition adjusted to the manufacturer's recommendations (Figure 5). In one test of the salt‐active nuclease, a salt‐tolerant protease from the same manufacturer replaced the otherwise used proteinase K.

The obtained titers from the AAV‐containing lysate sample after digest with each nuclease were generally comparable to the same AAV sample not treated with a nuclease. An apparent slightly lower titer for nucleases Denarase and M‐San HQ hints at a possible challenge to inactivate these enzymes. Future optimizations could explore the utility of a reduced amount of enzyme to reduce over‐digestion or inactivation problems. In contrast, incomplete digestion, as tested with the no‐AAV production control (no pRepCap was transfected), was evident only after DNaseI digest, showing that all other tested nucleases are principally suitable for digestion within 3 h. For this work, digestion of crude cell lysate with SAN‐HQ and AZ‐Protease proved effective and was used.

## 4 Discussion

There is growing demand to improve rAAV production systems for yield and homogeneity. However, the yield of an rAAV production process may be deeply rooted in AAV biology, which is finetuned to its host.

Interestingly, we found that AAV packaging plasmids in circulation today harbor changes to the Rep gene which are often not discussed in the respective publications. These include the location, modification and truncation of the p5 promoter and modifications to the Rep78/52 Kozak sequence with a weak start codon and an associated substitution of proline at position 2 with alanine. Such a change to the Kozak sequence has been discussed in context of the baculovirus production system, where toxic overexpression from the Rep78 open reading frame was reduced by the weak start codon to improve production. As the Kozak consensus sequence reaches beyond the start codon to +1, the P2A substitution was introduced [33]. However, most plasmids analyzed here, mainly from the Addgene repository, were clearly meant for production in mammalian cells and the almost full‐factorial set of combinations of the various p5 and Rep modifications without side‐by‐side comparison poses challenges in judging their universal usefulness.

In our three‐plasmid production setting in HEK‐293 cells, the rAAV2 titer increased dramatically when we reverted the Rep gene back to the wild‐type configuration. Although we cannot comment on all production settings (e.g., other producer cells, stable cell lines, and other plasmid configurations), the vast benefit of the wildtype p5 promoter, albeit still located upstream of the plasmid backbone, and natural Rep Kozak sequence in our setting means that the Rep configuration can be an important lever, when a production setting performs unexpectedly poorly.

The final rAAV2 titer in our test was close to 10^6^ vg per transfected cell, which is remarkably similar, if not higher, to what has been reported for wild‐type AAV2 (1.3 × 10^5^ vg/cell) [34] or another parvovirus, Minute Virus of Mice (7 × 10^5^) [35], although limited data are available. Apparently, wild‐type productivity has been restored, or surpassed, even with a recombinant production based on AAV2, which is often described as a low‐producer [36]. Although a productivity per cell is not always published in rAAV production engineering literature, from our understanding, a limit in the ballpark of 10^6^ per cell appears to be the current “ceiling productivity” and additional changes to the production system may be additive in effect, but the overall yield currently approaches this ceiling asymptotically.

The AAV life cycle was early on believed to be in mutual symbiosis with the human host [37]. Various examples of mutual or conditionally mutual virus‐host relationships are known in the literature [38]. One could derive the hypothesis that the AAV titer is evolutionary restrained to maintain the mutual relationship, and, therefore, probably also in the HEK‐293 recombinant production setting that mimics a productive infection in many aspects. Owing to its complex coding and function, the Rep proteins are largely resisting rational engineering to address this question. Explorative approaches based on saturation mutagenesis [5] or family shuffling [6] showed limited success in production improvement. In line with these studies, we did not find an improvement in viral genome titer from rational mutagenesis of Rep. However—and previously unexplored—the empty capsid titer varied dramatically in our experiments, suggesting that genome replication remains the rate‐limiting step, while the empty capsid titer may be tuned to this limit by means of Rep engineering.

One approach toward better understanding of the AAV production process is simplification. We, therefore, created a mini Ad5 helper plasmid, pHelper‐mini, with only three genetic elements of Ad5: DBP, E4 34k (E4orf6) and the VA‐RNAs. This pHelper‐mini initially lead to overexpression of E4 34k and downregulation of Rep, and an overall reduced titer. The titer could be mostly rescued for AAV2 by reduction of the pHelper‐mini gene dose, but not for AAV6 and AAV9 (both with AAV2‐Rep, a suboptimal AAV2‐Rep in case of AAV6), suggesting a role of the Rep68/40 N‐terminus overlapping Cap, a role for AAP6/9, MAAP6/9 or capsid specific challenges to titer in relation with the helper functions. Other studies have previously investigated Ad5 mini helper plasmids [9, 10, 39]. Interestingly, our pHelper‐mini lacks the L4 22k CDS which was identified as important in the previous studies, but still produces AAV2 to high titers. These findings are not necessarily contradictory, but highlight the complexity of the system and many possible levers that can be pulled, with individual changes contributing only incrementally to the final titer. For example, the Rep configuration and titer per transfected cell is not given in the previous publications and small changes may contribute.

We conclude that current AAV production systems can produce AAV at high titers when all their genetic elements are properly configured. Additional increases of the yield may be possible, but probably require a more in‐depth knowledge of AAV virology or entirely new approaches. At 10^6^ vg/cell, the AAV yield is already far surpassing cellular yields of other viral vectors and wild‐type viruses (e.g., the particularly productive SARS‐CoV‐2 is reported to produce about 10^5^ viruses per cell at peak infection [40]). Radically new approaches to AAV‐based therapies, like cell‐free production of AAV capsids [41, 42, 43] combined with in‐vitro packaging may provide additional avenues for safe and effective gene therapies to all who need them.

## Author Contributions

Kristian M. Müller, Marco T. Radukic, Dinh To Le, Claire Rothschild‐Gronau, Rebecca C. Feiner, and Kathrin E. Teschner conceptualized the study. Marco T. Radukic, Dinh To Le, Robert Freudenberg, Anne Hammann, Omar Hamdan, and Susanne K. Golm performed investigations. Raimund Hoffrogge contributed methodology and data curation for mass spectrometry. Marco T. Radukic contributed formal analysis, software, visualization, and wrote the original draft. All authors contributed to review and editing. Kristian M. Müller supervised the project.

## Funding

This research received no external funding.

## Conflicts of Interest

The authors declare no conflicts of interest.

## Supporting information




**Supporting Information file 1**: biot70179‐sup‐0001‐SuppMat.docx

## Data Availability

Data are available within the article.
