# Supplementary material for: Engineering of High‐Yield Recombinant Adeno‐Associated Virus Producer Plasmids
Source: Biotechnol J. 2026 Jan 14;21(1):e70179. doi: 10.1002/biot.70179 (PMC12802539; doi:10.1002/biot.70179)
Supplement: Supplementary file 1 — Supporting Information file 1: biot70179‐sup‐0001‐SuppMat.docx [file BIOT-21-e70179-s001.docx]

Supplementary information for

Engineering of high-yield recombinant adeno-associated virus producer plasmids

Marco T. Radukic^1^, Dinh To Le^1^, Robert Freudenberg^1^, Anne Hammann^1^, Omar Hamdan^1^, Claire Rothschild-Gronau^1^, Raimund Hoffrogge^2^, Susanne K. Golm^1^, Rebecca C. Feiner^1^, Kathrin E. Teschner^1^, and Kristian M. Müller^1,*^

^1^Cellular and Molecular Biotechnology, Bielefeld University, Faculty of Technology, Bielefeld, Germany

^2^Center for Biotechnology (CeBiTec), Bielefeld University, Bielefeld, Germany

*****Correspondence: kristian@syntbio.net

**Supplementary sequence S1: pZMB0430**

Packaging plasmid encoding Rep2 Cap2. Like pZMB216 with added XmaI-site (overlap-extension PCR) downstream of the Rep CDS though a silent Cap mutation to facilitate cloning of Rep variants; introduces a L22P mutation in MAAP, progenitor to plasmids pZMB0431 – pZMB0440.

AAAAGGCCGCGTTGCTGGCGTTTTTCCATAGGCTCCGCCCCCCTGACGAGCATCACAAAAATCGACGCTCAAGTCAGAGGTGGCGAAACCCGACAGGACTATAAAGATACCAGGCGTTTCCCCCTGGAAGCTCCCTCGTGCGCTCTCCTGTTCCGACCCTGCCGCTTACCGGATACCTGTCCGCCTTTCTCCCTTCGGGAAGCGTGGCGCTTTCTCATAGCTCACGCTGTAGGTATCTCAGTTCGGTGTAGGTCGTTCGCTCCAAGCTGGGCTGTGTGCACGAACCCCCCGTTCAGCCCGACCGCTGCGCCTTATCCGGTAACTATCGTCTTGAGTCCAACCCGGTAAGACACGACTTATCGCCACTGGCAGCAGCCACTGGTAACAGGATTAGCAGAGCGAGGTATGTAGGCGGTGCTACAGAGTTCTTGAAGTGGTGGCCTAACTACGGCTACACTAGAAGGACAGTATTTGGTATCTGCGCTCTGCTGAAGCCAGTTACCTTCGGAAAAAGAGTTGGTAGCTCTTGATCCGGCAAACAAACCACCGCTGGTAGCGGTGGTTTTTTTGTTTGCAAGCAGCAGATTACGCGCAGAAAAAAAGGATCTCAAGAAGATCCTTTGATCTTTTCTACGGGGTCTGACGCTCAGTGGAACGAAAACTCACGTTAAGGGATTTTGGTCATGAGATTATCAAAAAGGATCTTCACCTAGATCCTTTTAAATTAAAAATGAAGTTTTAAATCAATCTAAAGTATATATGAGTAAACTTGGTCTGACAGCTCGAGGCTTGGATTCTCACCAATAAAAAACGCCCGGCGGCAACCGAGCGTTCTGAACAAATCCAGATGGAGTTCTGAGGTCATTACTGGATCTATCAACAGGAGTCCAAGCGAGCTCGATATCAAATTACGCCCCGCCCTGCCACTCATCGCAGTACTGTTGTAATTCATTAAGCATTCTGCCGACATGGAAGCCATCACAAACGGCATGATGAACCTGAATCGCCAGCGGCATCAGCACCTTGTCGCCTTGCGTATAGTATTTGCCCATGGTGAAAACGGGGGCGAAGAAGTTGTCCATATTGGCCACGTTTAAATCAAAACTGGTGAAACTCACCCAGGGATTGGCTGAGACGAAAAACATATTCTCAATAAACCCTTTAGGGAAATAGGCCAGGTTTTCACCGTAACACGCCACATCTTGCGAATATATGTGTAGAAACTGCCGGAAATCGTCGTGGTATTCACTCCAGAGCGATGAAAACGTTTCAGTTTGCTCATGGAAAACGGTGTAACAAGGGTGAACACTATCCCATATCACCAGCTCACCGTCTTTCATTGCCATACGAAATTCCGGATGAGCATTCATCAGGCGGGCAAGAATGTGAATAAAGGCCGGATAAAACTTGTGCTTATTTTTCTTTACGGTCTTTAAAAAGGCCGTAATATCGAGCTGAACGGTCTGGTTATAGGTACATTGAGCAACTGACTGAAATGCCTCAAAATGTTCTTTACGATGCCATTGGGATATATCAACGGTGGTATATCCAGTGATTTTTTTCTCCATTTTAGCTTCCTTAGCTCCTGAAAATCTCGATAACTCAAAAAATACGCCCGGTAGTGATCTTATTTCATTATGGTGAAAGTTGGAACCTCTTACGTGCCCGATCAACTCGAGTGCCACTTGACGTCTAAGAAACCATTATTATCATGACATTAACCTATAAAAATAGGCGTATCACGAGGCAGAATTTCAGATAAAAAAAATCCTTAGCTTTCGCTAAGGATGATTTCTGGAATTCGCGGCCGCTTCTAGATGGCGGGGTTTTACGAGATTGTGATTAAGGTCCCCAGCGACCTTGACGAGCATCTGCCCGGCATTTCTGACAGCTTTGTGAACTGGGTGGCCGAGAAGGAATGGGAGTTGCCGCCAGATTCTGACATGGATCTGAATCTGATTGAGCAGGCACCCCTGACCGTGGCCGAGAAGCTCCAGCGCGACTTTCTGACGGAATGGCGCCGTGTGAGTAAGGCCCCGGAGGCCCTTTTCTTTGTGCAATTTGAGAAGGGAGAGAGCTACTTCCACATGCACGTGCTCGTGGAAACCACCGGGGTGAAATCCATGGTTTTGGGACGTTTCCTGAGTCAGATTCGCGAAAAACTGATTCAGAGAATTTACCGCGGGATCGAGCCGACTTTGCCAAACTGGTTCGCGGTCACAAAGACCAGAAATGGCGCCGGAGGCGGGAACAAGGTGGTGGATGAGTGCTACATCCCCAATTACTTGCTCCCCAAAACCCAGCCTGAGCTCCAGTGGGCGTGGACTAATATGGAACAGTATTTAAGCGCCTGTTTGAATCTCACGGAGCGTAAACGGTTGGTGGCGCAGCATCTGACGCACGTGTCGCAGACGCAGGAGCAGAACAAAGAGAATCAGAATCCCAATTCTGATGCGCCGGTGATCAGATCAAAAACTTCAGCCAGGTACATGGAGCTGGTCGGGTGGCTCGTGGACAAGGGGATTACCTCGGAGAAGCAGTGGATTCAGGAGGACCAGGCCTCATACATCTCCTTCAATGCGGCCTCCAACTCGCGGTCCCAAATCAAGGCTGCCTTGGACAATGCGGGAAAGATTATGAGCCTGACTAAAACCGCCCCCGACTACCTGGTGGGCCAGCAGCCCGTGGAGGACATTTCCAGCAATCGGATTTATAAAATTTTGGAACTAAACGGGTACGATCCCCAATATGCGGCTTCCGTCTTTCTGGGATGGGCCACGAAAAAGTTCGGCAAGAGGAACACCATCTGGCTGTTTGGGCCTGCAACTACCGGGAAGACCAACATCGCGGAGGCCATAGCCCACACTGTGCCCTTCTACGGGTGCGTAAACTGGACCAATGAGAACTTTCCCTTCAACGACTGTGTGGACAAGATGGTGATCTGGTGGGAGGAGGGGAAGATGACCGCCAAGGTCGTGGAGTCGGCCAAAGCCATTCTCGGAGGAAGCAAGGTGCGCGTGGACCAGAAATGCAAGTCCTCGGCCCAGATAGACCCGACTCCCGTGATCGTCACCTCCAACACCAACATGTGCGCCGTGATTGACGGGAACTCAACGACCTTCGAACACCAGCAGCCGTTGCAAGACCGGATGTTCAAATTTGAACTCACCCGCCGTCTGGATCATGACTTTGGGAAGGTCACCAAGCAGGAAGTCAAAGACTTTTTCCGGTGGGCAAAGGATCACGTGGTTGAGGTGGAGCATGAGTTCTACGTCAAAAAGGGTGGAGCCAAGAAAAGACCCGCCCCCAGTGACGCAGATATAAGTGAGCCCAAACGGGTGCGCGAGTCAGTTGCGCAGCCATCGACGTCAGACGCGGAAGCTTCGATCAACTACGCAGACAGGTACCAAAACAAATGTTCTCGTCACGTGGGCATGAATCTGATGCTGTTTCCCTGTAGACAATGCGAGAGAATGAATCAGAACTCAAATATCTGCTTCACTCACGGACAGAAAGACTGTTTAGAGTGCTTTCCCGTGTCAGAATCTCAACCCGTTTCTGTCGTCAAAAAGGCGTATCAGAAACTGTGCTACATTCATCATATCATGGGAAAGGTGCCAGACGCTTGCACTGCCTGCGATCTGGTCAATGTGGATTTGGATGACTGCATCTTTGAACAATAAATGATTTAAATCAGGTATGGCTGCCGATGGTTATCTTCCAGATTGGCTCGAGGACACTCTCTCTGAAGGAATAAGACAGTGGTGGAAGCTCAAACCTGGCCCACCACCACCAAAGCCCGCAGAGCGGCATAAGGACGACAGCAGGGGTCTTGTGCTTCCCGGGTACAAGTACCTCGGACCCTTCAACGGACTCGACAAGGGAGAGCCGGTCAACGAGGCAGACGCCGCGGCCCTCGAGCACGACAAAGCCTACGACCGGCAGCTCGACAGCGGAGACAACCCGTACCTCAAGTACAACCACGCCGACGCGGAGTTTCAGGAGCGCCTTAAAGAAGATACGTCTTTTGGGGGCAACCTCGGACGAGCAGTCTTCCAGGCGAAAAAGAGGGTTCTTGAACCTCTGGGCCTGGTTGAGGAACCTGTTAAGACGGCTCCGGGAAAAAAGAGGCCGGTAGAGCACTCTCCTGTGGAGCCAGACTCCTCCTCGGGAACCGGAAAGGCGGGCCAGCAGCCTGCAAGAAAAAGATTGAATTTTGGTCAGACTGGAGACGCAGACTCAGTACCTGACCCCCAGCCTCTCGGACAGCCACCAGCAGCCCCCTCTGGTCTGGGAACTAATACGATGGCTACAGGCAGTGGCGCACCAATGGCAGACAATAACGAGGGCGCCGACGGAGTGGGTAATTCCTCGGGAAATTGGCATTGCGATTCCACATGGATGGGCGACAGAGTCATCACCACCAGCACCCGAACCTGGGCCCTGCCCACCTACAACAACCACCTCTACAAACAAATTTCCAGCCAATCAGGAGCCTCGAACGACAATCACTACTTTGGCTACAGCACCCCTTGGGGGTATTTTGACTTCAACAGATTCCACTGCCACTTTTCACCACGTGACTGGCAAAGACTCATCAACAACAACTGGGGATTCCGACCCAAGAGACTCAACTTCAAGCTCTTTAACATTCAAGTCAAAGAGGTCACGCAGAATGACGGTACGACGACGATTGCCAATAACCTTACCAGCACGGTTCAGGTGTTTACTGACTCGGAGTACCAGCTCCCGTACGTCCTCGGCTCGGCGCATCAAGGATGCCTCCCGCCGTTCCCAGCAGACGTCTTCATGGTGCCACAGTATGGATACCTCACCCTGAACAACGGGAGTCAGGCAGTAGGACGCTCTTCATTTTACTGCCTGGAGTACTTTCCTTCTCAGATGCTGCGTACCGGAAACAACTTTACCTTCAGCTACACTTTTGAGGACGTTCCTTTCCACAGCAGCTACGCTCACAGCCAGAGTCTGGACCGTCTCATGAATCCTCTCATCGACCAATATTTGTATTACTTGAGCAGAACAAACACTCCAAGTGGTACCACCACGCAGAGTCGACTTCAGTTTTCTCAGGCCGGAGCGAGTGACATTCGGGACCAGTCTAGGAACTGGCTTCCTGGACCCTGTTACCGCCAGCAGCGAGTATCAAAGACATCTGCGGATAACAACAACAGTGAATACTCGTGGACTGGAGCTACCAAGTACCACCTCAATGGCAGAGACTCTCTGGTGAATCCGGGCCCGGCCATGGCAAGCCACAAGGACGATGAAGAAAAGTTTTTTCCTCAGAGCGGGGTTCTCATCTTTGGGAAGCAAGGCTCAGAGAAAACAAATGTGGACATTGAAAAGGTCATGATTACAGACGAAGAGGAAATCAGGACAACCAATCCCGTGGCTACGGAGCAGTATGGATCCGTATCTACCAACCTCCAGAGAGGCAACAGACAAGCAGCTACAGCTGATGTCAACACACAAGGCGTTCTTCCAGGCATGGTCTGGCAGGACAGAGATGTGTACCTTCAGGGGCCCATCTGGGCAAAGATTCCACACACGGACGGACATTTTCACCCCTCTCCCCTCATGGGTGGATTCGGACTTAAACACCCTCCTCCACAGATTCTCATCAAGAACACCCCGGTACCTGCGAATCCTTCGACCACCTTCAGTGCGGCAAAGTTTGCTTCCTTCATCACACAGTACTCCACGGGACAGGTCAGCGTGGAGATCGAGTGGGAGCTCCAGAAGGAAAACAGCAAACGCTGGAATCCCGAAATTCAGTACACTTCCAACTACAACAAGTCTGTTAATGTGGACTTTACTGTGGACACTAATGGCGTGTATTCAGAGCCTCGCCCCATTGGCACCAGATACCTGACTCGTAATCTGTAATTGCTTGTTAATCAATAAACCGTTTAATTCGTTTCAGTTGAACTTTGGTCTCTGCGTATTTCTTTCTTATCTAGTTTCCATGGCTACGTAGATAAGTAGCATGGCGGGTTAATCATTAACTACTACTAGAGGGAGGGGTGGAGTCGTGACGTGAATTACGTCATAGGGTTAGGGAGGTCCTGTATTAGAGGTCACGTGAGTGTTTTGCGACATTTTGCGACACCATGTGGTCTCGCTGGGGGGGGGGGCCCGAGTGAGCACGCAGGGTCTCCATTTTGAAGCGGGAGGTTTGAACTACTAGTAGCGGCCGCTGCAGTCCGGCAAAAAAACGGGCAAGGTGTCACCACCCTGCCCTTTTTCTTTAAAACCGAAAAGATTACTTCGCGTTATGCAGGCTTCCTCGCTCACTGACTCGCTGCGCTCGGTCGTTCGGCTGCGGCGAGCGGTATCAGCTCACTCAAAGGCGGTAATACGGTTATCCACAGAATCAGGGGATAACGCAGGAAAGAACATGTGAGCAAAAGGCCAGCAAAAGGCCAGGAACCGTA

**Supplementary sequence S2: pZMB0797**

pHelper-mini. Compared to an Ad5 reference, GenBank AC_000008, retained parts were for VA-RNA, roughly nucleotides 10,436 – 11,167, for E4 roughly nucleotides 34,087 – 33,195, and for E2A roughly nucleotides 24,055 – 22,188 with polymorphisms retained from the Agilent pHelper, pZMB0088. The plasmid was ordered as a gene synthesis.

TACGGGGTCTGACGCTCAGTGGAACGAAAACTCACGTTAAGGGATTTTGGTCATGAGATTATCAAAAAGGATCTTCACCTAGATCCTTTTAAATTAAAAATGAAGTTTTAAATCAATCTAAAGTATATATGAGTAAACTTGGTCTGACAGTTATTAGAAAAATTCATCCAGCAGACGATAAAACGCAATACGCTGGCTATCCGGTGCCGCAATGCCATACAGCACCAGAAAACGATCCGCCCATTCGCCGCCCAGTTCTTCCGCAATATCACGGGTGGCCAGCGCAATATCCTGATAACGATCCGCCACGCCCAGACGGCCGCAATCAATAAAGCCGCTAAAACGGCCATTTTCCACCATAATGTTCGGCAGGCACGCATCACCATGGGTCACCACCAGATCTTCGCCATCCGGCATGCTCGCTTTCAGACGCGCAAACAGCTCTGCCGGTGCCAGGCCCTGATGTTCTTCATCCAGATCATCCTGATCCACCAGGCCCGCTTCCATACGGGTACGCGCACGTTCAATACGATGTTTCGCCTGATGATCAAACGGACAGGTCGCCGGGTCCAGGGTATGCAGACGACGCATGGCATCCGCCATAATGCTCACTTTTTCTGCCGGCGCCAGATGGCTAGACAGCAGATCCTGACCCGGCACTTCGCCCAGCAGCAGCCAATCACGGCCCGCTTCGGTCACCACATCCAGCACCGCCGCACACGGAACACCGGTGGTGGCCAGCCAGCTCAGACGCGCCGCTTCATCCTGCAGCTCGTTCAGCGCACCGCTCAGATCGGTTTTCACAAACAGCACCGGACGACCCTGCGCGCTCAGACGAAACACCGCCGCATCAGAGCAGCCAATGGTCTGCTGCGCCCAATCATAGCCAAACAGACGTTCCACCCACGCTGCCGGGCTACCCGCATGCAGGCCATCCTGTTCAATCATACTCTTCCTTTTTCAATATTATTGAAGCATTTATCAGGGTTATTGTCTCATGAGCGGATACATATTTGAATGTATTTAGAAAAATAAACAAATAGGGGTTCCGCGCACATTTCCCCGAAAAGTGCCACCTAAATTGTAAGCGTTAATATTTTGTTAAAATTCGCGTTAAATTTTTGTTAAATCAGCTCATTTTTTAACCAATAGGCCGAAATCGGCAAAATCCCTTATAAATCAAAAGAATAGACCGAGATAGGGTTGAGTGGCCGCTACAGGGCGCTCCCATTCGCCATTCAGGCTGCGCAACTGTTGGGAAGGGCGTTTCGGTGCGGGCCTCTTCGCTATTACGCCAGCTGGCGAAAGGGGGATGTGCTGCAAGGCGATTAAGTTGGGTAACGCCAGGGTTTTCCCAGTCACGACGTTGTAAAACGACGGCCAGTGAGCGCGACGTAATACGACTCACTATAGGGCGAATTGAAGGAAGGCCGTCAAGGCCGCATGAGCTCATCCGGTACCCAACTCCATGCTTAACAGTCCCCAGGTACAGCCCACCCTGCGTCGCAACCAGGAACAGCTCTACAGCTTCCTGGAGCGCCACTCGCCCTACTTCCGCAGCCACAGTGCGCAGATTAGGAGCGCCACTTCTTTTTGTCACTTGAAAAACATGTAAAAATAATGTACTAGGAGACACTTTCAATAAAGGCAAATGTTTTTATTTGTACACTCTCGGGTGATTATTTACCCCCCACCCTTGCCGTCTGCGCCGTTTAAAAATCAAAGGGGTTCTGCCGCGCATCGCTATGCGCCACTGGCAGGGACACGTTGCGATATTGGTGTTTAGTGCTCCACTTAAACTCAGGCACAACCATCCGCGGCAGCTCGGTGAAGTTTTCACTCCACAGGCTGCGCACCATCACCAACGCGTTTAGCAGGTCGGGCGCCGATATCTTGAAGTCGCAGTTGGGGCCTCCGCCCTGCGCGCGCGAGTTGCGATACACAGGGTTGCAGCACTGGAACACTATCAGCGCCGGGTGGTGCACGCTGGCCAGCACGCTCTTGTCGGAGATCAGATCCGCGTCCAGGTCCTCCGCGTTGCTCAGGGCGAACGGAGTCAACTTTGGTAGCTGCCTTCCCAAAAAGGGTGCATGCCCAGGCTTTGAGTTGCACTCGCACCGTAGTGGCATCAGAAGGTGACCGTGCCCGGTCTGGGCGTTAGGATACAGCGCCTGCATGAAAGCCTTGATCTGCTTAAAAGCCACCTGAGCCTTTGCGCCTTCAGAGAAGAACATGCCGCAAGACTTGCCGGAAAACTGATTGGCCGGACAGGCCGCGTCATGCACGCAGCACCTTGCGTCGGTGTTGGAGATCTGCACCACATTTCGGCCCCACCGGTTCTTCACGATCTTGGCCTTGCTAGACTGCTCCTTCAGCGCGCGCTGCCCGTTTTCGCTCGTCACATCCATTTCAATCACGTGCTCCTTATTTATCATAATGCTCCCGTGTAGACACTTAAGCTCGCCTTCGATCTCAGCGCAGCGGTGCAGCCACAACGCGCAGCCCGTGGGCTCGTGGTGCTTGTAGGTTACCTCTGCAAACGACTGCAGGTACGCCTGCAGGAATCGCCCCATCATCGTCACAAAGGTCTTGTTGCTGGTGAAGGTCAGCTGCAACCCGCGGTGCTCCTCGTTTAGCCAGGTCTTGCATACGGCCGCCAGAGCTTCCACTTGGTCAGGCAGTAGCTTGAAGTTTGCCTTTAGATCGTTATCCACGTGGTACTTGTCCATCAACGCGCGCGCAGCCTCCATGCCCTTCTCCCACGCAGACACGATCGGCAGGCTCAGCGGGTTTATCACCGTGCTTTCACTTTCCGCTTCACTGGACTCTTCCTTTTCCTCTTGCGTCCGCATACCCCGCGCCACTGGGTCGTCTTCATTCAGCCGCCGCACCGTGCGCTTACCTCCCTTGCCGTGCTTGATTAGCACCGGTGGGTTGCTGAAACCCACCATTTGTAGCGCCACATCTTCTCTTTCTTCCTCGCTGTCCACGATCACCTCTGGGGATGGCGGGCGCTCGGGCTTGGGAGAGGGGCGCTTCTTTTTCTTTTTGGACGCAATGGCCAAATCCGCCGTCGAGGTCGATGGCCGCGGGCTGGGTGTGCGCGGCACCAGCGCATCTTGTGACGAGTCTTCTTCGTCCTCGGACTCGAGACGCCGCCTCAGCCGCTTTTTTGGGGGCGCGCGGGGAGGCGGCGGCGACGGCGACGGGGACGACACGTCCTCCATGGTTGGTGGACGTCGCGCCGCACCGCGTCCGCGCTCGGGGGTGGTTTCGCGCTGCTCCTCTTCCCGACTGGCCATTTCCTTCTCCTATAGGCAGAGGTACTGGTGTCCGCAGCTGCCTGTATCACAAAAGCGAAGATCAGCTTCGGCGCACGCTGGAAGACGCGGAGGCTCTCTTCAGCAAATACTGCGCGCTGACTCTTAAGGACTAGTTTCGCGCCCTTTCTCAAATTTAAGCGCGAAAACTACGTCATCTCCAGCGGCCACACCCGGCGCCAGCACCTGTCGTCAGCGCCATTATGAGCAAGGAAATTCCCACGCCCTACATGTGGAGTTACCAGCCACAAATGGGACTTGCGGCTGGAGCTGCCCAAGACTACTCAACCCGAATAAACTACATGAGCGCGGGACCCCACATGATATCCCGGGTCAACGGAATCCGCGCCCACCGAAACCGAATTCTCCTCGAACAGGCGGCTATTACCACCACACCTCGTAATAACCTTAATCCCCGTAGTTGGCCCGCTGCCCTGGTGTACCAGGAAAGTCCCGCTCCCACCACTGTGGTACTTCCCAGAGACGCCCAGGCCGAAGTTCAGATGACTAACTCAGGGGCGCAGCTTGCGGGCGGCTTTCGTCACAGGGTGCGGTCAAGCTTGCCCGGGCGTTTTAGGGCGGAGTAACTTGCATGTATTGGGAATTGTAGTTTTTTTAAAATGGGAAGTGACGTATCGTGGGAAAACGGAAGTGAAGATTTGAGGAAGTTGTGGGTTTTTTGGCTTTCGTTTCTGGGCGTAGGTTCGCGTGCGGTTTTCTGGGTGTTTTTTGTGGACTTTAACCGTTACGTCATTTTTTAGTCCTATATATACTCGCTCTGTACTTGGCCCTTTTTACACTGTGACTGATTGAGCTGGTGCCGTGTCGAGTGGTGTTTTTTAATAGGTTTTTTTACGAAGAGCATCAAGTCAAGCTCTTCACTGGTTTTGCTTCAGGAAATATGACTACGTCCGGCGTTCCATTTGGCATGACACTACGACCAACACGATCTCGGTTGTCTCGGCGCACTCCGTACAGTAGGGATCGCCTACCTCCTTTTGAGACAGAGACCCGCGCTACCATACTGGAGGATCATCCGCTGCTGCCCGAATGTAACACTTTGACAATGCACAACGTGAGTTACGTGCGAGGTCTTCCCTGCAGTGTGGGATTTACGCTGATTCAGGAATGGGTTGTTCCCTGGGATATGGTTCTGACGCGGGAGGAGCTTGTAATCCTGAGGAAGTGTATGCACGTGTGCCTGTGTTGTGCCAACATTGATATCATGACGAGCATGATGATCCATGGTTACGAGTCCTGGGCTCTCCACTGTCATTGTTCCAGTCCCGGTTCCCTGCAGTGCATAGCCGGCGGGCAGGTTTTGGCCAGCTGGTTTAGGATGGTGGTGGATGGCGCCATGTTTAATCAGAGGTTTATATGGTACCGGGAGGTGGTGAATTACAACATGCCAAAAGAGGTAATGTTTATGTCCAGCGTGTTTATGAGGGGTCGCCACTTAATCTACCTGCGCTTGTGGTATGATGGCCACGTGGGTTCTGTGGTCCCCGCCATGAGCTTTGGATACAGCGCCTTGCACTGTGGGATTTTGAACAATATTGTGGTGCTGTGCTGCAGTTACTGTGCTGATTTAAGTGAGATCAGGGTGCGCTGCTGTGCCCGGAGGACAAGGCGTCTCATGCTGCGGGCGGTGCGAATCATCGCTGAGGAGACCACTGCCATGTTGTATTCCTGCAGGACGGAGCGGCGGCGGCAGCAGTTTATTCGCGCGCTGCTGCAGCACCACCGCCCTATCCTGATGCACGATTATGACTCTACCCCCATGTAGGCGTGGAGAAGCTTGACTACGTTTGATTAAGGTACGGTGATCAATATAAGCTATGTGGTGGTGGGGCTATACTACTGAATGAAAAATGACTTGAAATTTTCTGCAATTGAAAAATAAACACGTTGAAACATAACATGCAACAGGTTCACGATTCTTTATTCCTGGGCAATGTAGGAGAAGGTGTAAGAGTTGGTAGCAAAAGTTTCAGTGGTGTATTTTCCACTTTCCCAGGACCATGTAAAAGACATAGAGTAAGTGCTTACCTCGCTAGTTTCTGTGGATTCACTAGAAGGCGCGCCATCGATGTAGGATGTTGCCCCTCCTGACGCGGTAGGAGAAGGGGAGGGTGCCCTGCATGTCTGCCGCTGCTCTTGCTCTTGCCGCTGCTGAGGAGGGGGGCGCATCTGCCGCAGCACCGGATGCATCTGGGAAAAGCAAAAAAGGGGCTCGTCCCTGTTTCCGGAGGAATTTGCAAGCGGGGTCTTGCATGACGGGGAGGCAAACCCCCGTTCGCCGCAGTCCGGCCGGCCCGAGACTCGAACCGGGGGTCCTGCGACTCAACCCTTGGAAAATAACCCTCCGGCTACAGGGAGCGAGCCACTTAATGCTTTCGCTTTCCAGCCTAACCGCTTACGCCGCGCGCGGCCAGTGGCCAAAAAAGCTAGCGCAGCAGCCGCCGCGCCTGGAAGGAAGCCAAAAGGAGCGCTCCCCCGTTGTCTGACGTCGCACACCTGGGTTCGACACGCGGGCGGTAACCGCATGGATCACGGCGGACGGCCGGATCCGGGGTTCGAACCCCGGTCGTCCGCCATGATACCCTTGCGAATTTATCCACCAGACCACGGAAGAGTGCCCGCTTACAGGCTCTCCTTTTGCACGGTCTAGAGCGTCAACGACTGCGCACGCCTCACCGGCCAGAGCGTCCCGACCATGGAGCACTTTTTGCCGCTGCGCAACATCTGGAACCGCGTCCGCGACTTTCCGCGCGCCTCCACCACCGCCGCCGGCATCACCTGGATGTCCAGGTACATCTACGGATTACGTCGACGTTTAAACCATATGCTGGGCCTCATGGGCCTTCCTTTCACTGCCCGCTTTCCAGTCGGGAAACCTGTCGTGCCAGCTGCATTAACATGGTCATAGCTGTTTCCTTGCGTATTGGGCGCTCTCCGCTTCCTCGCTCACTGACTCGCTGCGCTCGGTCGTTCGGGTAAAGCCTGGGGTGCCTAATGAGCAAAAGGCCAGCAAAAGGCCAGGAACCGTAAAAAGGCCGCGTTGCTGGCGTTTTTCCATAGGCTCCGCCCCCCTGACGAGCATCACAAAAATCGACGCTCAAGTCAGAGGTGGCGAAACCCGACAGGACTATAAAGATACCAGGCGTTTCCCCCTGGAAGCTCCCTCGTGCGCTCTCCTGTTCCGACCCTGCCGCTTACCGGATACCTGTCCGCCTTTCTCCCTTCGGGAAGCGTGGCGCTTTCTCATAGCTCACGCTGTAGGTATCTCAGTTCGGTGTAGGTCGTTCGCTCCAAGCTGGGCTGTGTGCACGAACCCCCCGTTCAGCCCGACCGCTGCGCCTTATCCGGTAACTATCGTCTTGAGTCCAACCCGGTAAGACACGACTTATCGCCACTGGCAGCAGCCACTGGTAACAGGATTAGCAGAGCGAGGTATGTAGGCGGTGCTACAGAGTTCTTGAAGTGGTGGCCTAACTACGGCTACACTAGAAGAACAGTATTTGGTATCTGCGCTCTGCTGAAGCCAGTTACCTTCGGAAAAAGAGTTGGTAGCTCTTGATCCGGCAAACAAACCACCGCTGGTAGCGGTGGTTTTTTTGTTTGCAAGCAGCAGATTACGCGCAGAAAAAAAGGATCTCAAGAAGATCCTTTGATCTTTTC

**Supplementary sequence S3: pZMB0916**

Packaging plasmid encoding Rep2 and Cap2. Similar to variant C, main text Fig. 1, Kozak and p5 wild-type, but with added ΔMAAP.

TCTTTTCTACGGGGTCTGACGCTCAGTGGAACGAAAACTCACGTTAAGGGATTTTGGTCATGAGATTATCAAAAAGGATCTTCACCTAGATCCTTTTAAATTAAAAATGAAGTTTTAAATCAATCTAAAGTATATATGAGTAAACTTGGTCTGACAGTTACCAATGCTTAATCAGTGAGGCACCTATCTCAGCGATCTGTCTATTTCGTTCATCCATAGTTGCCTGACTCCCCGTCGTGTAGATAACTACGATACGGGAGGGCTTACCATCTGGCCCCAGTGCTGCAATGATACCGCGAGACCCACGCTCACCGGCTCCAGATTTATCAGCAATAAACCAGCCAGCCGGAAGGGCCGAGCGCAGAAGTGGTCCTGCAACTTTATCCGCCTCCATCCAGTCTATTAATTGTTGCCGGGAAGCTAGAGTAAGTAGTTCGCCAGTTAATAGTTTGCGCAACGTTGTTGCCATTGCTACAGGCATCGTGGTGTCACGCTCGTCGTTTGGTATGGCTTCATTCAGCTCCGGTTCCCAACGATCAAGGCGAGTTACATGATCCCCCATGTTGTGCAAAAAAGCGGTTAGCTCCTTCGGTCCTCCGATCGTTGTCAGAAGTAAGTTGGCCGCAGTGTTATCACTCATGGTTATGGCAGCACTGCATAATTCTCTTACTGTCATGCCATCCGTAAGATGCTTTTCTGTGACTGGTGAGTACTCAACCAAGTCATTCTGAGAATAGTGTATGCGGCGACCGAGTTGCTCTTGCCCGGCGTCAATACGGGATAATACCGCGCCACATAGCAGAACTTTAAAAGTGCTCATCATTGGAAAACGTTCTTCGGGGCGAAAACTCTCAAGGATCTTACCGCTGTTGAGATCCAGTTCGATGTAACCCACTCGTGCACCCAACTGATCTTCAGCATCTTTTACTTTCACCAGCGTTTCTGGGTGAGCAAAAACAGGAAGGCAAAATGCCGCAAAAAAGGGAATAAGGGCGACACGGAAATGTTGAATACTCATACTCTTCCTTTTTCAATATTATTGAAGCATTTATCAGGGTTATTGTCTCATGAGCGGATACATATTTGAATGTATTTAGAAAAATAAACAAATAGGGGTTCCGCGCACATTTCCCCGAAAAGTGCCACCTAAATTGTAAGCGTTAATATTTTGTTAAAATTCGCGTTAAATTTTTGTTAAATCAGCTCATTTTTTAACCAATAGGCCGAAATCGGCAAAATCCCTTATAAATCAAAAGAATAGACCGAGATAGGGTTGAGTGTTGTTCCAGTTTGGAACAAGAGTCCACTATTAAAGAACGTGGACTCCAACGTCAAAGGGCGAAAAACCGTCTATCAGGGCGATGGCCCACTACGTGAACCATCACCCTAATCAAGTTTTTTGGGGTCGAGGTGCCGTAAAGCACTAAATCGGAACCCTAAAGGGAGCCCCCGATTTAGAGCTTGACGGGGAAAGCCGGCGAACGTGGCGAGAAAGGAAGGGAAGAAAGCGAAAGGAGCGGGCGCTAGGGCGCTGGCAAGTGTAGCGGTCACGCTGCGCGTAACCACCACACCCGCCGCGCTTAATGCGCCGCTACAGGGCGCGTCCCATTCGCCATTCAGGCTGCGCAACTGTTGGGAAGGGCGATCGGTGCGGGCCTCTTCGCTATTACGCCAGCTGGCGAAAGGGGGATGTGCTGCAAGGCGATTAAGTTGGGTAACGCCAGGGTTTTCCCAGTCACGACGTTGTAAAACGACGGCCAGTGAGCGCGCGTAATACGACTCACTATAGGGCGAATTGGGTACCGGGCCCCCCCTCGATCGAGGTCGACGGTATCGGGGGAGCTCGCAGGGTCTCCATTTTGAAGCGGGAGGTTTGAACGCGCAGCCGCCATGCCGGGGTTTTACGAGATTGTGATTAAGGTCCCCAGCGACCTTGACGAGCATCTGCCCGGCATTTCTGACAGCTTTGTGAACTGGGTGGCCGAGAAGGAATGGGAGTTGCCGCCAGATTCTGACATGGATCTGAATCTGATTGAGCAGGCACCCCTGACCGTGGCCGAGAAGCTGCAGCGCGACTTTCTGACGGAATGGCGCCGTGTGAGTAAGGCCCCGGAGGCTCTTTTCTTTGTGCAATTTGAGAAGGGAGAGAGCTACTTCCACATGCACGTGCTCGTGGAAACCACCGGGGTGAAATCCATGGTTTTGGGACGTTTCCTGAGTCAGATTCGCGAAAAACTGATTCAGAGAATTTACCGCGGGATCGAGCCGACTTTGCCAAACTGGTTCGCGGTCACAAAGACCAGAAATGGCGCCGGAGGCGGGAACAAGGTGGTGGATGAGTGCTACATCCCCAATTACTTGCTCCCCAAAACCCAGCCTGAGCTCCAGTGGGCGTGGACTAATATGGAACAGTATTTAAGCGCCTGTTTGAATCTCACGGAGCGTAAACGGTTGGTGGCGCAGCATCTGACGCACGTGTCGCAGACGCAGGAGCAGAACAAAGAGAATCAGAATCCCAATTCTGATGCGCCGGTGATCAGATCAAAAACTTCAGCCAGGTACATGGAGCTGGTCGGGTGGCTCGTGGACAAGGGGATTACCTCGGAGAAGCAGTGGATCCAGGAGGACCAGGCCTCATACATCTCCTTCAATGCGGCCTCCAACTCGCGGTCCCAAATCAAGGCTGCCTTGGACAATGCGGGAAAGATTATGAGCCTGACTAAAACCGCCCCCGACTACCTGGTGGGCCAGCAGCCCGTGGAGGACATTTCCAGCAATCGGATTTATAAAATTTTGGAACTAAACGGGTACGATCCCCAATATGCGGCTTCCGTCTTTCTGGGATGGGCCACGAAAAAGTTCGGCAAGAGGAACACCATCTGGCTGTTTGGGCCTGCAACTACCGGGAAGACCAACATCGCGGAGGCCATAGCCCACACTGTGCCCTTCTACGGGTGCGTAAACTGGACCAATGAGAACTTTCCCTTCAACGACTGTGTCGACAAGATGGTGATCTGGTGGGAGGAGGGGAAGATGACCGCCAAGGTCGTGGAGTCGGCCAAAGCCATTCTCGGAGGAAGCAAGGTGCGCGTGGACCAGAAATGCAAGTCCTCGGCCCAGATAGACCCGACTCCCGTGATCGTCACCTCCAACACCAACATGTGCGCCGTGATTGACGGGAACTCAACGACCTTCGAACACCAGCAGCCGTTGCAAGACCGGATGTTCAAATTTGAACTCACCCGCCGTCTGGATCATGACTTTGGGAAGGTCACCAAGCAGGAAGTCAAAGACTTTTTCCGGTGGGCAAAGGATCACGTGGTTGAGGTGGAGCATGAGTTCTACGTCAAAAAGGGTGGAGCCAAGAAAAGACCCGCCCCCAGTGACGCAGATATAAGTGAGCCCAAACGGGTGCGCGAGTCAGTTGCGCAGCCATCGACGTCAGACGCGGAAGCTTCGATCAACTACGCAGACAGGTACCAAAACAAATGTTCTCGTCACGTGGGCATGAATCTGATGCTGTTTCCCTGTAGACAATGCGAGAGAATGAATCAGAACTCAAATATCTGCTTCACTCACGGACAGAAAGACTGTTTAGAGTGCTTTCCCGTGTCAGAATCTCAACCCGTTTCTGTCGTCAAAAAGGCGTATCAGAAACTGTGCTACATTCATCATATCATGGGAAAGGTGCCAGACGCTTGCACTGCCTGCGATCTGGTCAATGTGGATTTGGATGACTGCATCTTTGAACAATAAATGATTTAAATCAGGTATGGCTGCCGATGGTTATCTTCCAGATTGGCTCGAGGACACTCTCTCTGAAGGAATAAGACAGTGGTGGAAGCTCAAACCGGGCCCACCACCACCAAAGCCCGCAGAGCGGCATAAGGACGACAGCAGGGGTCTTGTGCTTCCTGGGTACAAGTACCTCGGACCCTTCAACGGACTCGACAAGGGAGAGCCGGTCAACGAGGCAGACGCCGCGGCCCTCGAGCACGACAAAGCCTACGACCGGCAGCTCGACAGCGGAGACAACCCGTACCTCAAGTACAACCACGCCGACGCGGAGTTTCAGGAGCGCCTTAAAGAAGATACGTCTTTTGGGGGCAACCTCGGACGAGCAGTCTTCCAGGCGAAAAAGAGGGTTCTTGAACCTCTGGGCCTGGTTGAGGAACCTGTTAAGACGGCTCCGGGAAAAAAGAGGCCGGTAGAGCACTCTCCTGTGGAGCCAGACTCCTCCTCGGGAACCGGAAAGGCGGGCCAGCAGCCTGCAAGAAAAAGATTGAATTTTGGTCAGACTGGAGACGCAGACTCAGTACCTGACCCCCAGCCTCTCGGACAGCCACCAGCAGCCCCCTCTGGTCTGGGAACTAATACGATGGCTACAGGCAGTGGCGCACCAATGGCAGACAATAACGAGGGCGCCGACGGAGTGGGTAATTCCTCGGGAAATTGGCATTGCGATTCCACATGGATGGGCGACAGAGTCATCACCACCAGCACCCGAACCTGGGCCCTGCCCACCTACAACAACCACCTCTACAAACAAATTTCCAGCCAATCAGGAGCCTCGAACGACAATCACTACTTTGGCTACAGCACCCCTTGGGGGTATTTTGACTTCAACAGATTCCACTGCCACTTTTCACCACGTGACTGGCAAAGACTCATCAACAACAACTGGGGATTCCGACCCAAGAGACTCAACTTCAAGCTCTTTAACATTCAAGTCAAAGAGGTCACGCAGAATGACGGTACGACGACGATTGCCAATAACCTTACCAGCACGGTTCAGGTGTTTACTGACTCGGAGTACCAGCTCCCGTACGTCCTCGGCTCGGCGCATCAAGGATGCCTCCCGCCGTTCCCAGCAGACGTCTTCATGGTGCCACAGTATGGATACCTCACCCTGAACAACGGGAGTCAGGCAGTAGGACGCTCTTCATTTTACTGCCTGGAGTACTTTCCTTCTCAGATGCTGCGTACCGGAAACAACTTTACCTTCAGCTACACTTTTGAGGACGTTCCTTTCCACAGCAGCTACGCTCACAGCCAGAGTCTGGACCGTCTCATGAATCCTCTCATCGACCAATATTTGTATTACTTGAGCAGAACAAACACTCCAAGTGGTACCACCACGCAGAGTCGACTTCAGTTTTCTCAGGCCGGAGCGAGTGACATTCGGGACCAGTCTAGGAACTGGCTTCCTGGACCCTGTTACCGCCAGCAGCGAGTATCAAAGACATCTGCGGATAACAACAACAGTGAATACTCGTGGACTGGAGCTACCAAGTACCACCTCAATGGCAGAGACTCTCTGGTGAATCCGGGCCCGGCCATGGCAAGCCACAAGGACGATGAAGAAAAGTTTTTTCCTCAGAGCGGGGTTCTCATCTTTGGGAAGCAAGGCTCAGAGAAAACAAATGTGGACATTGAAAAGGTCATGATTACAGACGAAGAGGAAATCAGGACAACCAATCCCGTGGCTACGGAGCAGTATGGATCCGTATCTACCAACCTCCAGAGAGGCAACAGACAAGCAGCTACAGCTGATGTCAACACACAAGGCGTTCTTCCAGGCATGGTCTGGCAGGACAGAGATGTGTACCTTCAGGGGCCCATCTGGGCAAAGATTCCACACACGGACGGACATTTTCACCCCTCTCCCCTCATGGGTGGATTCGGACTTAAACACCCTCCTCCACAGATTCTCATCAAGAACACCCCGGTACCTGCGAATCCTTCGACCACCTTCAGTGCGGCAAAGTTTGCTTCCTTCATCACACAGTACTCCACGGGACAGGTCAGCGTGGAGATCGAGTGGGAGCTCCAGAAGGAAAACAGCAAACGCTGGAATCCCGAAATTCAGTACACTTCCAACTACAACAAGTCTGTTAATGTGGACTTTACTGTGGACACTAATGGCGTGTATTCAGAGCCTCGCCCCATTGGCACCAGATACCTGACTCGTAATCTGTAATTGCTTGTTAATCAATAAACCGTTTAATTCGTTTCAGTTGAACTTTGGTCTCTGCGTATTTCTTTCTTATCTAGTTTCCATGGCTACGTAGATAAGTAGCATGGCGGGTTAATCATTAACTACTACTAGAGGGAGGGGTGGAGTCGTGACGTGAATTACGTCATAGGGTTAGGGAGGTCCTGTATTAGAGGTCACGTGAGTGTTTTGCGACATTTTGCGACACCATGTGGTCTCGCTGGGTATTTAAGCCCGAGTGAGCACGCAGGGTCTCCATTTTGAAGCGGGAGGTTTGAACTACTAGTAGCGGCCGCTGCAGTCCGGCAAAAAAACGGGCAAGGTGTCACCACCCTGCCCTTTTTCTTTAAAACCGAAAAGATTACTTCGCGTTATGCAGGCTTCCTCGCTCACTGACTCGCTGCGCTCGGTCGTTCGGCTGCGGCGAGCGGTATCAGCTCACTCAAAGGCGGTAATACGGTTATCCACAGAATCAGGGGATAACGCAGGAAAGAACATGTGAGCAAAAGGCCAGCAAAAGGCCAGGAACCGTAAAAAGGCCGCGTTGCTGGCGTTTTTCCATAGGCTCCGCCCCCCTGACGAGCATCACAAAAATCGACGCTCAAGTCAGAGGTGGCGAAACCCGACAGGACTATAAAGATACCAGGCGTTTCCCCCTGGAAGCTCCCTCGTGCGCTCTCCTGTTCCGACCCTGCCGCTTACCGGATACCTGTCCGCCTTTCTCCCTTCGGGAAGCGTGGCGCTTTCTCATAGCTCACGCTGTAGGTATCTCAGTTCGGTGTAGGTCGTTCGCTCCAAGCTGGGCTGTGTGCACGAACCCCCCGTTCAGCCCGACCGCTGCGCCTTATCCGGTAACTATCGTCTTGAGTCCAACCCGGTAAGACACGACTTATCGCCACTGGCAGCAGCCACTGGTAACAGGATTAGCAGAGCGAGGTATGTAGGCGGTGCTACAGAGTTCTTGAAGTGGTGGCCTAACTACGGCTACACTAGAAGAACAGTATTTGGTATCTGCGCTCTGCTGAAGCCAGTTACCTTCGGAAAAAGAGTTGGTAGCTCTTGATCCGGCAAACAAACCACCGCTGGTAGCGGTGGTTTTTTTGTTTGCAAGCAGCAGATTACGCGCAGAAAAAAAGGATCTCAAGAAGATCCTTTGA
